# Mesenchymal stem cells derived from inflamed dental pulpal and gingival tissue: a potential application for bone formation

**DOI:** 10.1186/s13287-017-0633-z

**Published:** 2017-08-01

**Authors:** Laura Tomasello, Rodolfo Mauceri, Antonina Coppola, Maria Pitrone, Giuseppe Pizzo, Giuseppina Campisi, Giuseppe Pizzolanti, Carla Giordano

**Affiliations:** 1 Laboratory of Regenerative Medicine “Aldo Galluzzo”, Department of Endocrinology, Diabetology and Metabolism, University of Palermo, Piazza Delle Cliniche 2, 90127 Palermo, Italy; 2 Advanced Technologies Network Center, University of Palermo, Palermo, Italy; 3 Department of Surgical, Oncological and Oral Sciences, University of Palermo, Palermo, Italy

**Keywords:** Inflammation, Dental disease, Pulpal and gingival mesenchymal stem cells, Bone formation, Heat shock protein, ADFs, Proinflammatory cytokines

## Abstract

**Background:**

Chronic periodontal disease is an infectious disease consisting of prolonged inflammation of the supporting tooth tissue and resulting in bone loss. Guided bone regeneration procedures have become common and safe treatments in dentistry, and in this context dental stem cells would represent the ideal solution as autologous cells. In this study, we verified the ability of dental pulp mesenchymal stem cells (DPSCs) and gingival mesenchymal stem cells (GMSCs) harvested from periodontally affected teeth to produce new mineralized bone tissue in vitro, and compared this to cells from healthy teeth.

**Methods:**

To characterize DPSCs and GMSCs, we assessed colony-forming assay, immunophenotyping, mesenchymal/stem cell phenotyping, stem gene profiling by means of flow cytometry, and quantitative polymerase chain reaction (qPCR). The effects of proinflammatory cytokines on mesenchymal stem cell (MSC) proliferation and differentiation potential were investigated. We also observed participation of several heat shock proteins (HSPs) and actin-depolymerizing factors (ADFs) during osteogenic differentiation.

**Results:**

DPSCs and GMSCs were successfully isolated both from periodontally affected dental tissue and controls. Periodontally affected dental MSCs proliferated faster, and the inflamed environment did not affect MSC marker expressions. The calcium deposition was higher in periodontally affected MSCs than in the control group.

Proinflammatory cytokines activate a cytoskeleton remodeling, interacting with HSPs including HSP90 and HSPA9, thioredoxin-1, and ADFs such as as profilin-1, cofilin-1, and vinculin that probably mediate the increased acquisition in the inflamed environment.

**Conclusions:**

Our findings provide evidence that periodontally affected dental tissue (both pulp and gingiva) can be used as a source of MSCs with intact stem cell properties. Moreover, we demonstrated that the osteogenic capability of DPSCs and GMSCs in the test group was not only preserved but increased by the overexpression of several proinflammatory cytokine-dependent chaperones and stress response proteins.

**Electronic supplementary material:**

The online version of this article (doi:10.1186/s13287-017-0633-z) contains supplementary material, which is available to authorized users.

## Background

Chronic periodontal disease is an infectious disease resulting in inflammation within the supporting tissue of the tooth, with progressive attachment and bone loss. It is characterized by pocket formation and/or gingival recession [1]. Some 5% to 20% of any population suffers from severe, generalized periodontitis; mild to moderate periodontitis affects a majority of adults and represents the main cause of tooth loss [2]. Along with dental caries, periodontal disease is the main cause of tooth loss; the teeth most commonly lost due to periodontal problems are the first and the second molars in the maxilla [3]. As result of tooth loss, the alveolar process undergoes bone resorption, causing a reduction of the amount of available bone for the insertion of dental implants and the achievement of prosthetic rehabilitation [4, 5].

Guided bone regeneration (GBR) procedures have become a common and safe treatment in dentistry, and autografts are considered the gold standard in GBR procedures for their osteogenic and osteoinductive properties. However, the principal limits are that a donor site is required and only a limited amount of graft is often recoverable [6–8]. For this reason, autologous mesenchymal stem cells (MSCs) would represent the ideal solution for stem cell-based bone tissue engineering.

Tissue engineering (TE) and regenerative medicine (RM) are emerging fields, focused on the development of alternative strategies for tissue or organ repair, that have made significant progress in the last years [9, 10]. TE provides new regenerated tissues by the appliance of cells, scaffold, and growth factors, alone or in combination; nowadays RM has made exceptional progress leading to the regeneration of numerous organs and organ systems by using the capability of stem cells to differentiate into specialized cell types [11–13].

In adult humans stem cells are hosted in niches, a microenvironment that includes cellular and noncellular components that interact with each other to control the adult stem cells delegated to maintain the integrity of the tissues [14]. MSCs, defined as a population of nonhematopoietic fibroblast-like cells, able to differentiate into multiple lineages, including osteoblasts, adipocytes, and chondrocytes [9, 15]. During aging, the number of stem cell niches decreases, limiting the possibility of recognizing new sites for the collection of samples and obtaining multiple lines of differentiation for tissue engineering [14, 16, 17].

Over the last years, many niches have been described in the oral cavity: the dental pulp from permanent or deciduous teeth, the periodontal ligament, the apical papilla, the dental follicle, and the gingival tissue [18–22].

Recently, oral MSCs have also been harvested from dental tissue that is not healthy, such as fractured teeth and teeth affected by caries or irreversible pulpitis or aggressive periodontitis [23–26]. Dental pulp MSCs (DPSCs) and gingival MSCs (GMSCs) are clonogenic cells capable of both self-renewal and multiple lines of differentiation; moreover, compared to the bone marrow MSCs (BM-MSCs), DPSCs and GMSCs demonstrate the ability to proliferate faster, to be mostly homogenous, and to have excellent capacity to differentiate into osteogenic cells [18, 27–29]. It is controversial, however, whether proinflammatory cytokines could compromise multipotency and regenerative potential in several types of MSCs in vitro. There is growing evidence that proinflammatory cytokines such as interleukin (IL)-1β or tumor necrosis factor (TNF)-α are important causal factors of cellular proliferation and differentiation in human MSCs [26, 30–33]. Recently, some studies have focused on a possible link between proinflammatory cytokines, bone formation, and various heat shock proteins (HSPs). Inflammation and hypoxic conditions induce the expression of several HSPs engaged in protein folding and actin cytoskeletal organization [34–36]. The actin polymerization remodeling is a fundamental process during lineage-specific differentiation; in this context, Chen et al. showed that the inhibition of main actin depolymerizing factors (ADFs) enhance osteoblastic differentiation in human stromal stem cells [36].

In this study, we evaluated the inflammatory effects on human dental stem cells, particularly DPSCs and GMSCs from inflamed dental tissue, and we investigated if they can be used both as an MSC source and as host tissue in regenerative therapies. We compared the MSC markers, MSC gene profile, proliferation, and in vitro differentiation ability of the DPSCs and GMSCs harvested from periodontally compromised teeth compared to healthy teeth. We investigated if the proinflammatory microenvironment negatively affects dental MSC characteristics and properties, and we speculated about a closer link between chronic inflammation and bone formation through the involvement of several HSPs and ADFs.

## Methods

### Dental pulp and gingival tissue extraction

Dental pulp was extracted from the teeth of healthy adults aged 18–75 years. The eligibility criteria for participants were as follows: extraction needed for molars suffering from severe periodontal disease (mobility grade III; the test group), extraction needed for wisdom teeth for orthodontic reasons (the control group), no suspect or visibly pregnancy in females, and a positive response to the vitality test performed on teeth to be extracted.

Before the extraction, each patient had to rinse the mouth with 0.2% chlorhexidin for 1 min (Meridol®, Gaba Vebas S.r.l., Rome, Italy) to decontaminate the oral cavity. Gingival tissue was collected while the patient underwent oral surgery procedures for tooth extraction.

### Establishment of dental pulp and gingival cell cultures

After surgery, the pulpal or the gingival tissue was transferred in StemLine Mesenchymal Stem Cell Expansion Medium (Sigma-Aldrich, Milano, Italy) enriched with 0.2 mg/ml gentamicin, 0.25 mg/ml levofluoxacin, 0.10 mg/ml vancomicin, and 0.25 mg/ml fluconazole in a 50-ml tube, and within 24 h the samples were digested. The digestion was carried out in a solution of 5 mg/ml collagenase G (Abiel srl, Palermo, Italy) and 2 mg/ml collagenase H (Abiel srl, Palermo, Italy) in a 4:1 ratio for 4 h at 37 °C under agitation. The digests containing primary cells from the pulp or the gingiva were centrifuged and transferred to a T25 cell culture flask (EuroClone spa, Milano, Italy) or a p60 dish culture (referred to as passage (P)0). The cells were kept in culture in StemLine Mesenchymal Stem Cell Expansion Medium enriched with 0.5 μg/ml gentamicin, 0.25 μg/ml levofluoxacin, 0.10 μg/ml vancomicin, 0.25 μg/ml fluconazole, and 5% fetal bovine serum (FBS) and incubated at 37 °C in 5% CO_2_. Primary cells attached to the flask in 4–5 days; once they reached 80% confluence (in about 2 week) they were trypsinized and subculture was started (P1). By subculture P4, the antibiotic and antifungal cover was abolished (expansion medium). P1 to P8 cells were used for the in vitro assays.

### Colony-forming assay

A single-cell suspension (P0) of DPSCs and GMSCs from both the periodontally affected and healthy donors were seeded in a six-well culture in StemLine Mesenchymal Stem Cell Expansion Medium with 10% FBS at a density of 300 cells/well and cultured at 37 °C in 5% CO_2_. After 14 days, the cells were fixed in 4% paraformaldehyde and stained with 0.1% crystal violet. Only the cellular groups containing more than 50 cells were considered as colonies.

#### Population doubling, cell proliferation curve, and cytokine cytotoxicity

Proliferation was assayed by trypan blue (Sigma-Aldrich) according to the manufacturer’s instructions. The P2 GMSCs or P2 DPSCs from periodontally affected or healthy donors were seeded at a density of 4 × 10^3^ cells/cm^2^. The P4 healthy (H)-DPSCs or P4 H-GMSCs with or without 20 ng/ml IL-1β and 40 ng/ml TNF-α were seeded in a 96-well plate at a density of 4 × 110^3^ cells/cm^2^ and cultured up to 120 h. The cell counts were performed by optical microscope observation after trypan blue staining every 24 h during the incubation period. The doubling time (DT) was calculated accordingly to literature data (http://www.doubling-time.com/compute.php). Three sets of experiments for each sample were used for calculations.

#### Cytokine toxicity assay

The P4 H-GDPSCs or P4 H-DGMSCs with or without 20 ng/ml IL-1β and 40 ng/ml TFN-α were seeded in a 96-well plate at a density of 4 × 110^3^ cells/cm^2^ and cultured up to 72 h. The cell viability was evaluated by UV absorption at 550 nm at 24, 48, and 72 h using a microplate reader, after 3-(4,5-dimethylthiazol-2-yl)-2,5-diphenyltetrazolium bromide (MTT) incubation for 4 h at 37 °C. P5 BM-MSCs (Lonza, Walkersville, MD, USA) were used as the positive control.

### Flow cytometric analyses

#### Cell cycle cytofluorimetric analysis

Single-cell suspensions of periodontally affected (P)-DPSCs, H-DPSCs, P-GMSCs, and H-GMSCs (P4 culture passages) were obtained and DNA content analysis was performed according to Nicoletti’s protocol. Briefly, 1 × 10^6^ cells were fixed in 70% ethanol, rehydrated in phosphate-buffered saline (PBS), and then resuspended in a DNA extraction buffer (with 0.2 M NaHPO_4_ and 0.1% Tritonx-100 at pH 7.8). After staining with 1 μg/mL propidium iodide for 5 min, fluorescence intensity was determined by analysis on a FACS Calibur flow cytometer (Becton-Dickinson, New Jersey, USA). Data acquisition was performed with CellQuest (Becton Dickinson) software, and the percentages of G1, S, and G2 phase cells were calculated with the MODFIT-LT software program (Verity Software House, Inc.). The proliferation index (PI) was expressed as % G2 + % M.

#### Surface marker cytofluorimetric analysis

The P-DPSCs, H-DPSCs, P-GMSCs, and H-GMSCs (P4 culture passages) were harvested and filtered through a 40-μm filter mesh and suspended at a concentration of 1 × 110^6^ cells/ml. Then 100 μl of cell suspension containing 5 × 10^5^ cells was used for each flow cytometric test.

#### Immunophenotyping in flow cytometry

Human anti-HLA-DR, human anti-CD34, and human anti-CD45 monoclonal antibodies were tested on P4 P-DPSCs, H-DPSCs, P-GMSCs, and H-GMSCs, and were detected with the appropriate secondary antibody (Table 1). The incubation conditions were in accord with the manufacturer’s instructions. Unstained cells were used as the negative control. P4 BM-MSCs used as the positive control are not shown.

**Table 1:** Human antimonoclonal antibodies used in flow cytometry analysis for detection of mesenchymal stem cell markers

Antibody, localization marker	Code number	Dilution	Incubation
Primary antibodies			
Stro-1, surface	Thermo Fisher Sc, 39-8401	1:100	o/n, r.t.
CD146, surface	Milteny Biotec, 130-092-851	1:50	30 min, r.t
CD29, surface	Milteny Biotec, 130-101-258	1:50	30 min, r.t.
SSEA4, surface	Milteny Biotec, 130-98-371	1:100	30 min, r.t.
CD34, surface	Santa Cruz, sc-19621	1:50	o/n , r.t.
CD45, surface	Santa Cruz, sc-28369	1:50	o/n , r.t.
HLA-DR, surface	Santa Cruz , sc-18875	1:50	o/n , r.t.
Secondary antibody			
AlexaFluor 488	Life Technologies, Z25402	1:50	20 min, r.t.
AlexaFluor 594	Life Technologies, Z25007	1:50	20 min, r.t.

*o/n* overnight, *r.t.* room temperature

#### Stem cell phenotypes

The cells were tested for expression of the MSC surface markers Stro-1, CD146, CD29, and SSEA4, with the appropriate human anti-monoclonal antibody (Table 1). The antibody dilution, incubation, and detection conditions are also shown in Table 1.

All reaction mixtures were then acquired with a FACS Calibur flow cytometer (Becton-Dickinson, New Jersey, USA) and analyzed with the CellQuest Pro software. The specific isotype control antibodies were used as the negative control.

### Isolation of total RNA and polymerase chain reaction

Total RNA was extracted and purified using the E.Z.N.A. Total RNA Kit I (Omega Bio-Tek Inc., GA, USA) according to the manufacturer’s instructions. RNA quantity and quality were assessed by Nano Drop 2000 (Thermo Scientific); 2 μg limbal fibroblast-like stem cell (f-LSC) total RNA was reverse-transcribed to cDNA in a volume of 20 μl with Oligo dT primers (Applied Biosystems, CA, USA) and the Reverse Transcriptase Rnase kit (Improm II, Promega, WI, USA). Real-time quantitative polymerase chain reaction (qPCR) analyses were performed to analyze IL-1β receptor (IL-1β-R1) and TNF-α receptor (TNF-R1) expression, the cell proliferation, the stem gene profile, and the osteogenic differentiation, and to detect the expression of the ADFs and HSPs. All reactions were performed using the Quantitect SYBR Green PCR Kit (Qiagen, CA, USA) on the RotorGene Q Instrument (Qiagen). Each cDNA sample was mixed with specific primer sets (listed in Table 2) and PCR master mix. The qPCR reactions were performed using the following parameters for 45 cycles: denaturation at 95 °C for 3 min, 95 °C for 20 s, annealing at 60 °C for 30 s, and elongation at 72 °C for 60 s. Reactions were performed at least in triplicate. The specificity of the amplified products was determined by melting peak analysis. The relative quantification model with efficiency correction was applied to normalize the expression of the target gene to β-actin (used as the housekeeping gene) and to compare gene expression with BM-MSCs (used as a positive cell control) using the Delta Delta Ct method validated according to the guidelines of Livak and Schmittgen [37]. The results were represented as histograms on GraphPad Software by setting the gene expression of the positive control equal to 1. The MSCs were used at P5.

**Table 2:** The primer sequence list used for the amplification of mesenchymal stem cell cDNA

Gene	Primer sequence	Code number
ABCG2		QT00073206
CD105		QT0001335
THY-1		QT00023569
CD73		QT00027279
NANOG		QT01844808
OCT4		QT00210840
SOX2	*F:5'- GGAGACGGAGCTGAAGCCGC-3'* *R:5'GACGCGGTCCGGGCTTGTTTT-3'*	MWG
IL-1β-R1	*F:5'-CCAGGGAACTATTTTTATTTTCTGG-3'* *F:5'-CTGAGAAGCTGGACCCCTTG-3'*	MWG
TNF-R1	*F:5'-GGGATAAAAGGCAAAGACCAA-3'* *F:5'-TCCTTCACCGCTTCAGAAAA-3'*	MWG
ccnd1		QT00495285
cdkn1b		QT00998445
c-myc	*F:5'-AAACACAAACTTGAACAGCTAC-3'* *F:5'-ATTTGAGGCAGTTTACATTATGG-3'*	MWG
runx-2	*F:5'-TACGACTGGACGCTGGTGC-3'* *R:5'-TTCATGGGTCGCTTGACGT-3'*	MWG
opn	*F:5'-TGTGGGTTTCAGCACTCTGGTCA-3', R:5'-AAGCGAGTTGAATGGTGC-3'*	MWG
ocn	*F:5'-CTGACCTCACAGATGCCAAG-3'* *R:5'-GTAGCGCCGGAGTCTGTTC -3'*	
CFL-1 (cofilin)	*F:5'-TGCGGCTCCTACTAAACGG-3'* *F:5'-ACGCACCTTCATGTCGTTGA-3'*	MWG
PFN-1 (profilin)	*F:5'-ACCCGGAAACAAGAAGAC-3'* *F:5'-ACTGGTCCGATAACCTCCCA-3'*	MWG
VCL (vinculin)	*F:5'-ATGTCTCCTATATCCTGGTTT-3'* *F:5'-GCAGGAAGTGTCCTTCAGAC-3'*	MWG
HSPA9 (mortalin)	*F:5'-TACAGCAGATGGTGAGCGAC-3'* *R:5'-TGCTGTGTGCCCCAAGTAAT-3'*	MWG
TXN-1 (thioredoxin-1)	*F:5'- GTGAAGTCAAATGCACGCCA-3'* *R:5'-GCAGATGGCAACTGGTTATGT-3'*	MWG
HSP90AA1	*F:5'-GTGAAGTCAAATGCACGCCA-3'* *R:5'-GCAGATGGCAACTGGTTATGT-3'*	MWG

*F* forward, *R* reverse

### The protein-interaction networks (PIN)

Network analysis was performed on the ADFs, HSPs, and osteogenic proteins using the STRING (Search Tool for the Retrieval of Interacting Genes/Proteins) website (http://string-db.org/). The co-mentions, co-expression and associations in curated databeses were set as the evidence for functional links.

### In vitro bone formation

P-DPSCs, H-DPSCs plus cytokines, H-DPSCs, P-GMSCs, H-GMSCs plus cytokines, and H-GMSCs (all 5 × 10^3^/cm^2^) were cultured in home-made osteogenic differentiation medium (ODM); 5 × 10^3^/cm^2^ H-DPSCs and H-GMSCs were cytokine preconditioned. In detail, H-DPSCs plus cytokines and H-GMSCs plus cytokines were incubated up to 72 h in expansion medium with 20 ng/ml IL-1β and 40 ng/ml TNF-α and cultured in ODM. After 21 days of culture in the ODM, cells were stained with Alizarin Red S (Sigma-Aldrich, St. Louis, USA) to detect the calcium deposits. Briefly, the medium was removed and the cells were fixed with 4% formaldehyde solution for 30 min and, after fixation, rinsed twice with distilled water and stained with 2% Alizarin Red S (pH 4.2) for 3 min. After observation under a light optical microscope the images were acquired with a Nikon DS-fi1. The quantification of the calcium deposits was assessed by measurement of the optical density (OD) at 550 nm. ODM consisted of Dulbecco’s modified Eagle’s medium (DMEM) supplemented with 15% FBS, 10^–4^ mM dexamethasone (Sigma-Aldrich), 10 mM glycerophosphate (Sigma-Aldrich), and 0.05 mM ascorbic acid (Sigma-Aldrich) [9]. P6 MSCs were used for in vitro bone formation assay.

### Statistical analysis

All assays were performed in triplicate. The data are reported as means ± SD and compared using the appropriate version of the Student’s unpaired *t* test or one-way analysis of variance and post Tukey’s multiple comparison test. *p* < 0.05 was considered statistically significant.

## Results

### Inflamed dental tissue-derived MSCs show a higher proliferative ability

**Fig. 1:**
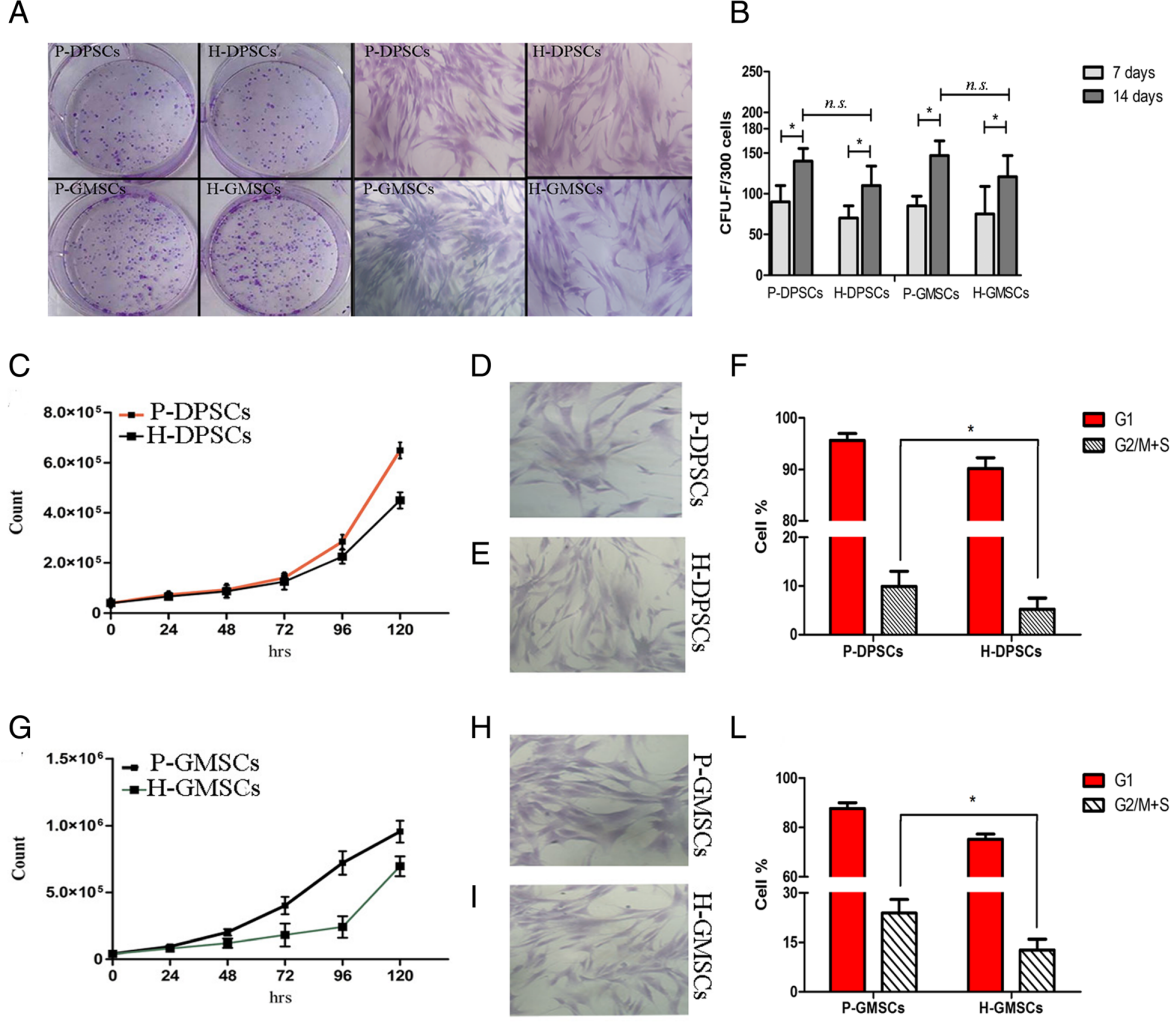
Colony-forming unit assays and monolayer subculture. **a** Representative images of pulpal (*DPSCs*) and gingival (*GMSCs*) mesenchymal stem cell colonies (P0) and monolayer subcultures (P1), isolated from periodontally affected (*P*) and healthy (*H*) patients and stained with crystal violet. **b** Colony-forming assay (*CFU*-*F*) in dental mesenchymal stem cells. **c** Cell growth curve of P-DPSCs and H-DPSCs by trypan blue viability assay (P2). **d**, **e** The typical fibroblast-like cell shape of a monolayer subculture of P-DPSCs and H-DPSCs. **f** The bar plot represents the comparative cell cycle distribution analysis between P-DPSCs and H-DPSC (G2M + S = proliferation index (PI)). **g** Cell growth curve of P-GMSCs and H-GMSCs by trypan blue viability assay. **h**, **i** The typical fibroblast-like cell shape of a monolayer subculture of P-GMSCs and H-GMSCs at 72 h. **l** The bar plot represents the comparative cell cycle distribution analysis between P-GMSCs and H-GMSC (G2M + S = PI). Cell cycle analysis at P4. *p < 0.05. *n.s.* not significant

DPSCs and GMSCs were isolated from 49 patients. The subjects were divided into two major groups: 1) the periodontally affected group (P, the test group; *n* = 37); and 2) the healthy group (H, the control group; *n* = 12). For each patient, pulpal and gingival tissues were extracted. Nine of the total samples (*n* = 5 test group; *n* = 4 control group) were unsuccessfully processed with high grades of bacterial contamination. For all 40 remaining samples, a cell suspension was generated after enzymatic digestion. The first plastic adherent cells were detected from all cultures between 7 and 10 days after preparation, and primary cells from healthy tissue initially grew much faster than those from periodontally affected tissue. The cultures (P0) appeared heterogeneous in shape and size, and the cells showed the ability to grow out from tissue now totally digested and to form clone-like growth (Fig. 1a). Generally, as culture progressed, gingival cells reached confluence at day 15 (12–18 days) and pulpal cells at day 20 (14–26). All the primary cells showed a typical fibroblast-like morphology, and they were homogeneous in shape and size (Fig. 1a). The CFU-F assay is the most frequently used test to analyze the clonogenic potential of isolated MSCs and to demonstrate MSC enrichment. Thus, we performed a CFU-F assay on all dental MSCs; P-DPSCs, H-DPSCs, P-GMSCs, and H-GMSCs displayed the highest time-dependent colony-forming ability, and a significant enrichment was observed in periodontally affected dental MSCs with respect to their healthy controls. Based on counting, the number of colonies per 300 seeded cells was 90.4 ± 18.07 and 140.2 ± 15.8 (*p* < 0.05) for the P-DPSCs at 7 and 14 days, respectively, and 88.3 ± 12.03 and 146.8 ± 28.8 (*p* < 0.05) for the P-GMSCs at 7 and 14 days, respectively; the results were 75.1 ± 15.05 and 121.2 ± 23.6 (*p* < 0.05) for the H-DPSCs at 7 and 14 days, respectively, and 70.3 ± 28.03 and 111.03 ± 24.8 (*p* < 0.05) for the H-GMSCs at 7 and 14 days, respectively (Fig. 1b). After they reached confluence, the cells were harvested and subcultured. From culture P1, a modest change in growth behavior was observed; in spite of the MTT assay revealing no significant difference (*p* > 0.05) in the growth rate (Additional file 1), the P-DPSCs and P-GMSCs proliferated faster than healthy control cells. DT was established at 28.83 ± 2 h vs. 34.37 ± 5 h for P-DPSCs vs. H-DPSCs, respectively, and at 26.22 ± 8 h vs. 29.13 ± 4 h for P-GMSCs vs. H-GMSCs, respectively (Fig. 1c and g; Additional file 1). The cell cycle analysis assigned a proliferation index (PI) G2M + S of 9.90 ± 3.1% vs. 5.23 ± 2.3%, respectively, in P-DPSCs vs. H-DPSCs and a PI of 23.85 ± 4.1% vs. 12.72 ± 3.24%, respectively, in P-GMSCs vs. H-GMSCs (Fig. 1f and l; Additional file 1). The difference in PI between P-DPSCs and H-DPSCs and between P-GMSCs and H-GMSCs was statistically significant (*p* < 0.005).

### Human healthy and periodontally affected gingival and pulpal dental cells express putative mesenchymal stem cell markers, are negative for hematopoietic differentiation clusters, and represent a stem gene profile

**Fig. 2:**
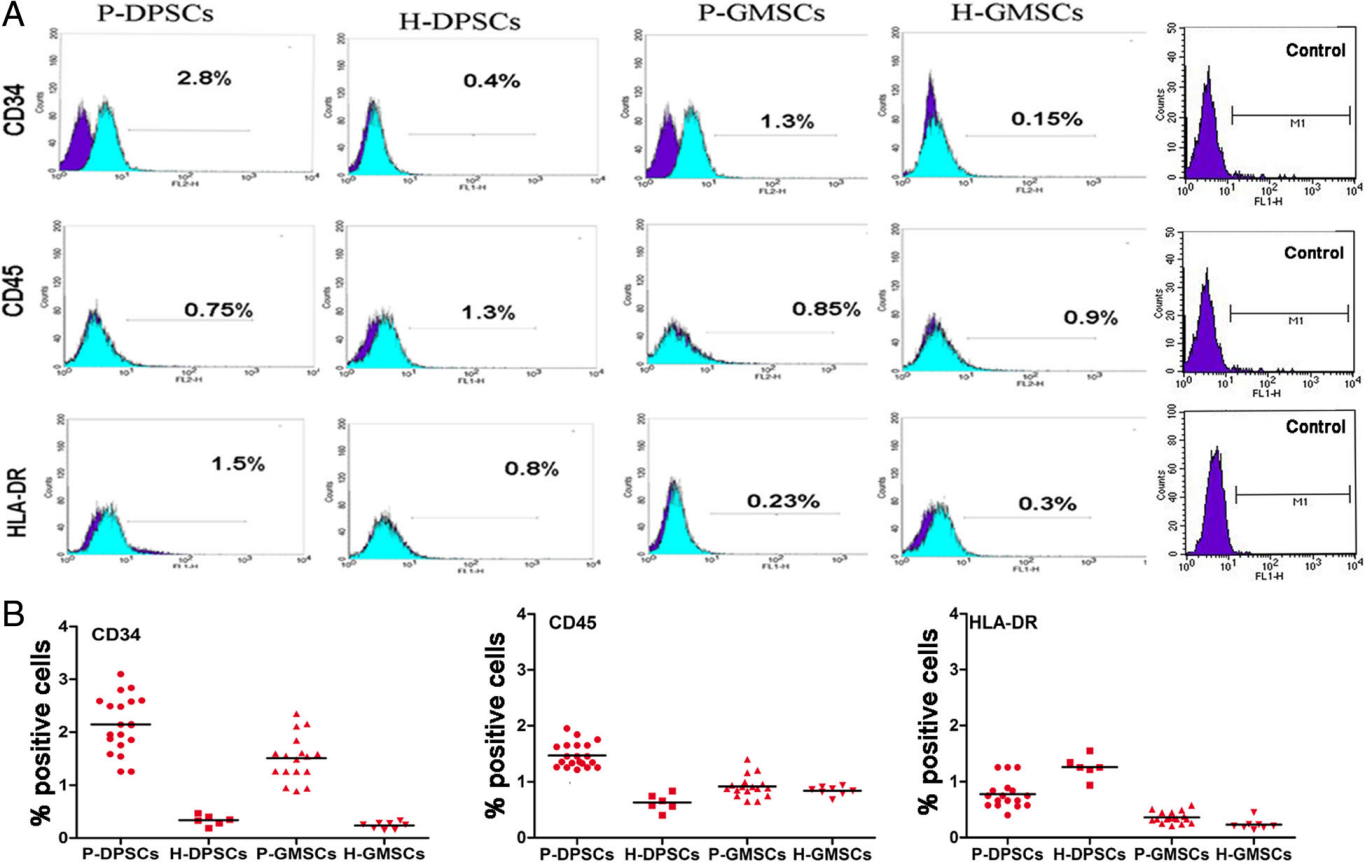
Immunophenotype flow cytometric assay*.* **a** Each field shows a representative sample. Cells are negative for CD34, CD45, and HLA-DR. The *green* peak shows the positive cells; the *purple* peak shows the isotype controls. The increases in CD34 in periodontally affected samples were not statistically significant. (Control: isotype controls were anti-IgM for Stro-1 and anti-IgG1 for SSEA4, CD146, and CD29.) **b** Scatter dot plots represent the expression levels of the immunophenotype cell markers (culture passage P5). *DPSC* dental pulp mesenchymal stem cell, *GMSC* gingival mesenchymal stem cell, *H* healthy donor, *P* periodontally affected donor

**Fig. 3:**
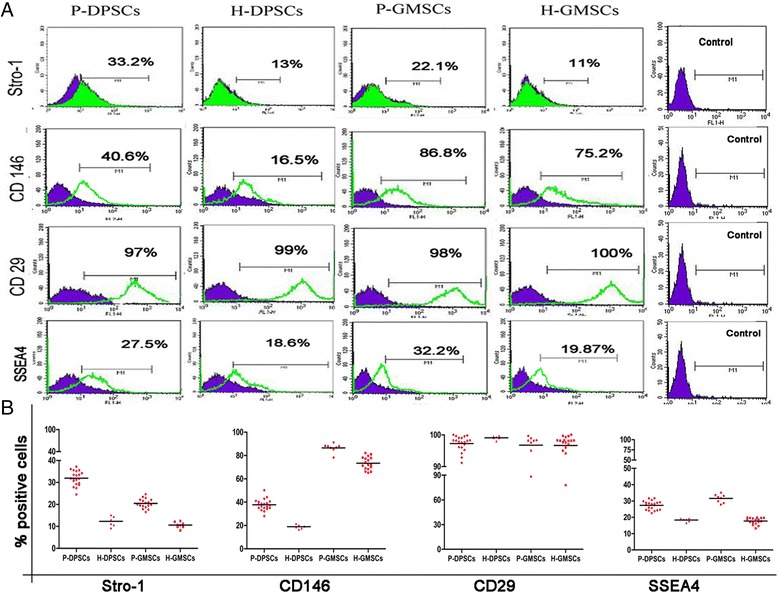
Stem cell phenotyping by flow cytometry. **a** A representative figure of the cytometry analysis in periodontally affected (*P*) pulpal (*DPSC*) or gingival (*GMSC*) mesenchymal stem cells (P-DPSCs and P-GMSCs, respectively) and their healthy controls (H-DPSCs and H-GMSCs). In the same histogram graph, the *green* peak shows the positive cells and the *purple* peak is the unstained cells used as negative control. (Control: Iisotype controls were anti-IgG1 for CD34 and CD45, and anti-IgG2 for HLA-DR.) **b** Scatter dot plot graphs represent expression levels of the mesenchymal stem cell markers Stro-1, CD146, CD29, and SSEA4 (culture passage P5)

The isolated cells did not display any hematopoietic surface markers (CD34 and CD45) and HLA-DR. Some increases were detected in the expression levels of CD34 in periodontally affected samples; however, this was not statistically significant (*p* > 0.005) (Fig. 2). The expression of putative the mesenchymal surface stem cells markers Stro-1, CD146, CD29, and SSEA4 was observed by flow cytometry and compared to BM-MSCs (data not shown) (Fig. 3). A higher Stro-1^+^/CD146^+^/SSEA-4^+^ cell population (*p* < 0.05) in P-DPSCs and P-GMSCs with respect to their healthy controls was detected. In all samples, CD29 stayed highly positive (about 100%). The expression values are reported in Additional file 2.

**Fig. 4:**
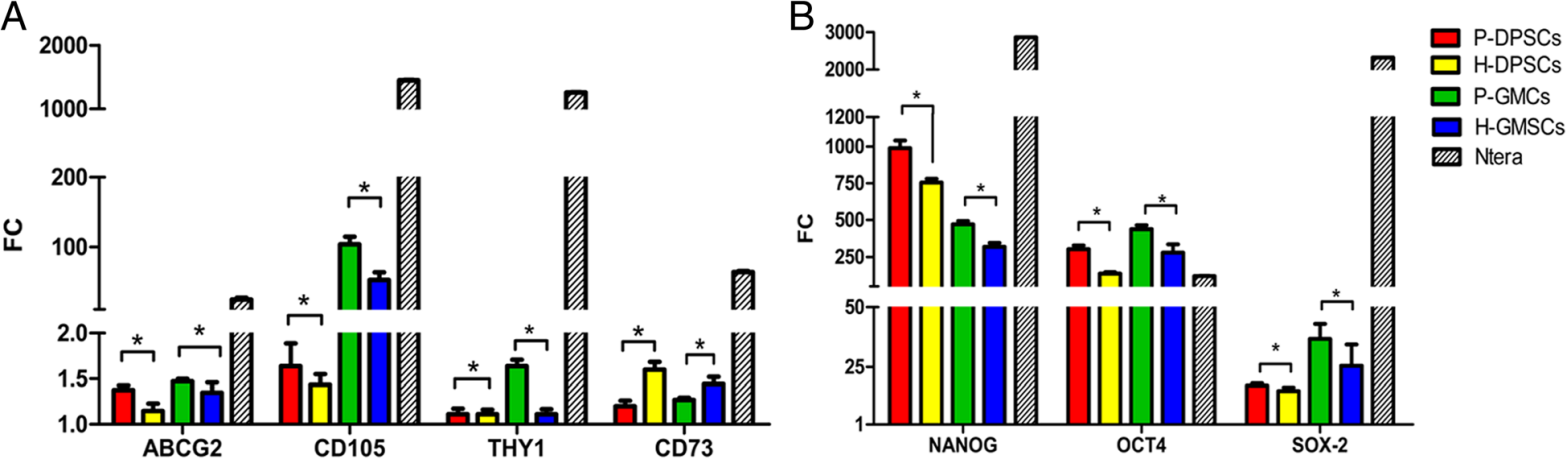
Stem cell gene expression profile of dental periodontally affected (*P*) and healthy (*H*) mesenchymal stem cells (*MSCs*) from dental pulp (*D*) and gingival (*G*) tissues. **a** Comparative analysis of surface marker expression in P-DPSCs, H-DPSCs, P-GMSCs, and H-GMSCs. **b** Comparative analysis of nuclear surface marker expression in P-DPSCs, H-DPSCs, P-GMSCs, and H-GMSCs. Mean values ± SD of all samples studied are reported. Actin β was used as the housekeeping gene. mRNA expression of all analyzed genes was normalized against BM-MSCs (positive control, relative gene expression value = 1). Culture passage P5. *p < 0.05; Ntera is used as internal control. *FC* fold change

We compared the stem cell molecular expression pattern in the P-DPSCs, P-GMSCs, H-DPSCs, and H-GMSCs as differences in fold change. Generally, we found a higher expression of all stem markers in dental mesenchymal stem cells with respect to the BM-MSCs (used as positive control; value of relative gene expression = 1). In detail, the mRNA levels of the embryonic stem cell markers NANOG and OCT4 showed very high fold change with respect to BM-MSCs. Moreover, the expression levels of the main stemness genes were significant higher in periodontally affected MSCs compared to their healthy controls whereas, a lower mRNA level of the CD73 surface marker was found in periodontally affected MSCs compared to their healthy control (*p*<0.05; Fig. 4).

### The proinflammatory cytokine cocktail facilitates pulpal and gingival mesenchymal stem cell expansion in vitro

**Fig. 5:**
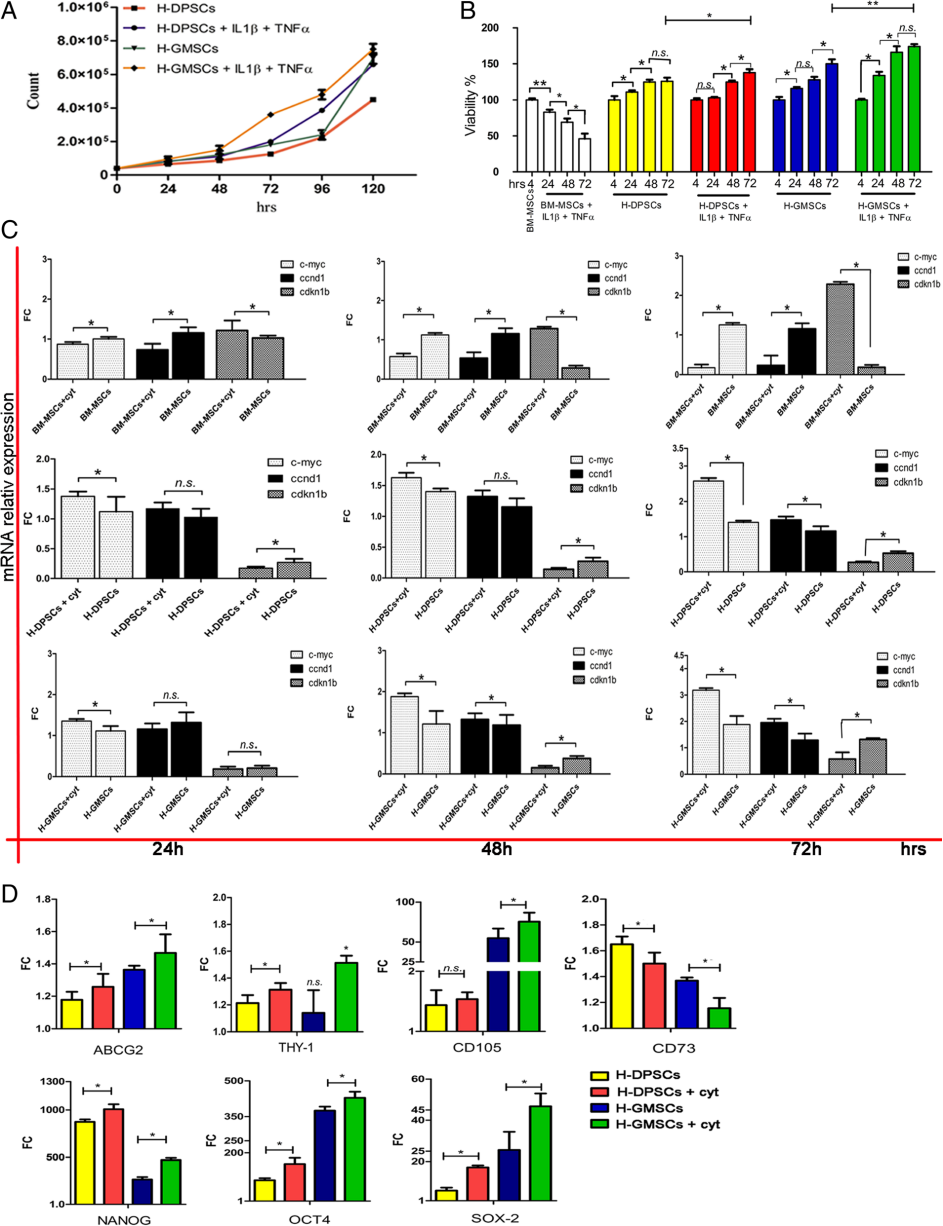
Two cytokines promote H-GMSC and H-DPSC expansion. **a** Cell growth curve of healthy dental pulp mesenchymal stem cells (*H-DPSCs*) and healthy gingival MSCs (*H-GMSCs*) with and without cytokines (*cyt*; 20 ng/ml interleukin-1beta (*IL1β*) and 40 ng/ml tumor necrosis factor alpha (*TNFα*) for 72 h) by trypan blue viability assay. **b** Cytotoxicity assay: H-DPSCs and H-GMSCs without and after cytokine treatment. **c** qPCR analysis of cell cycle regulators and proliferation markers ccnd1, cdnk1b, and c-myc in H-DPSCs + cyt and H-GMSCs + cyt and their healthy controls at 24, 48, and 72 h. **d** Comparative analysis of nuclear and surface marker expression in H-DPSCs + cyt and H-GMSCs + cyt and their healthy controls at 72 h. Bone marrow MSCs (*BM-MSCs*) used as positive control (relative gene expression value = 1); β-actin is the housekeeping gene. Mean values ± SD of all samples studied are reported. SD bars are based on three independent experiments. Culture passage P5. *p < 0.05. *FC* fold change, *n.s.* not significant

We investigated the effect of the proinflammatory cytokines IL-1β and TNF-α. Firstly, we confirmed the presence of IL-1β and TNF-α receptors in P-GMSCs and P-DPSCs and their healthy controls, in basal and under cytokine treatment conditions (Additional file 3). After 72 h of treatment no cytotoxic effect on cells was found; we even found a proliferation advantage acquisition. Indeed, a decrease in DT was detected in H-DPSCs and H-GMSCs treated with IL-1β and TNF-α, mimicking the P-DPSC and P-GMSC proliferation curve (Fig. 5a), and the MTT analysis showed an increase in the percentage of vital cells (Fig. 5b). We evaluated the principal molecules involved in cell proliferation. The qPCR analysis for c-myc (myc-protoncogene), ccnd1 (cyclin-D1), and cdkn1b (cyclin-dependent kinase inhibitor 1B) was assessed for each time point of culture in H-DPSCs and H-GMSCs treated with IL-1β and TNF-α versus untreated healthy controls. In line with the MTT results we found a significant upregulation of c-myc and ccnd1 in dental MSCs (Fig. 5c, middle and lower panels). Consistent with the above results, we found a downregulation of the ccnd1 inhibitor (cdkn1b). In addition, as a consequence of the cytokine treatment, the same genes were found to be modulated with the opposite trend in BM-MSCs (in Fig. 5c, upper panel).

#### Inflammation and stem cell gene profile correlation

To investigate whether the inflammation environment affects stem cell gene profile, a qPCR analysis was performed to compare H-GMSCs and H-DPSCs after 20 ng/ml IL-1β and 40 ng/ml TNF-α up to 72 h versus untreated H-GMSCs and H-DPSCs. In Fig. 5d the histograms represent the mRNA levels expressed as fold change. In cytokine-treated H-GMSCs and H-DPSCs all MSC markers showed an increase with respect to H-GMSCs and H-DPSCs (*p* < 0.05), except for CD105 in H-DPSCs after the treatment (p> 0.05) and CD73 which showed a decrease (*p* < 0.05).

We found increases of about 1.06- and 1.07-fold in ABCG2 (*p* < 0.05), 1.06 = and 1.37-fold in CD105 (*p* > 0.05 and *p* < 0.05), 1.08- and 1.33-fold in THY-1 (*p* < 0.05), 1.15- and 1.76-fold in NANOG (*p* < 0.05), 1.77- and 1.14-fold in OCT4 (*p* < 0.05), and 2.89- and 1.83-fold in SOX-2 (*p* < 0.05) in H-DPSCs plus cytokines vs H-DPSCs and H-GMSCs plus cytokines vs H-DPSCs, respectively.

#### In vitro bone formation: inflammation, cytoskeleton modulation, and osteogenesis

**Fig. 6:**
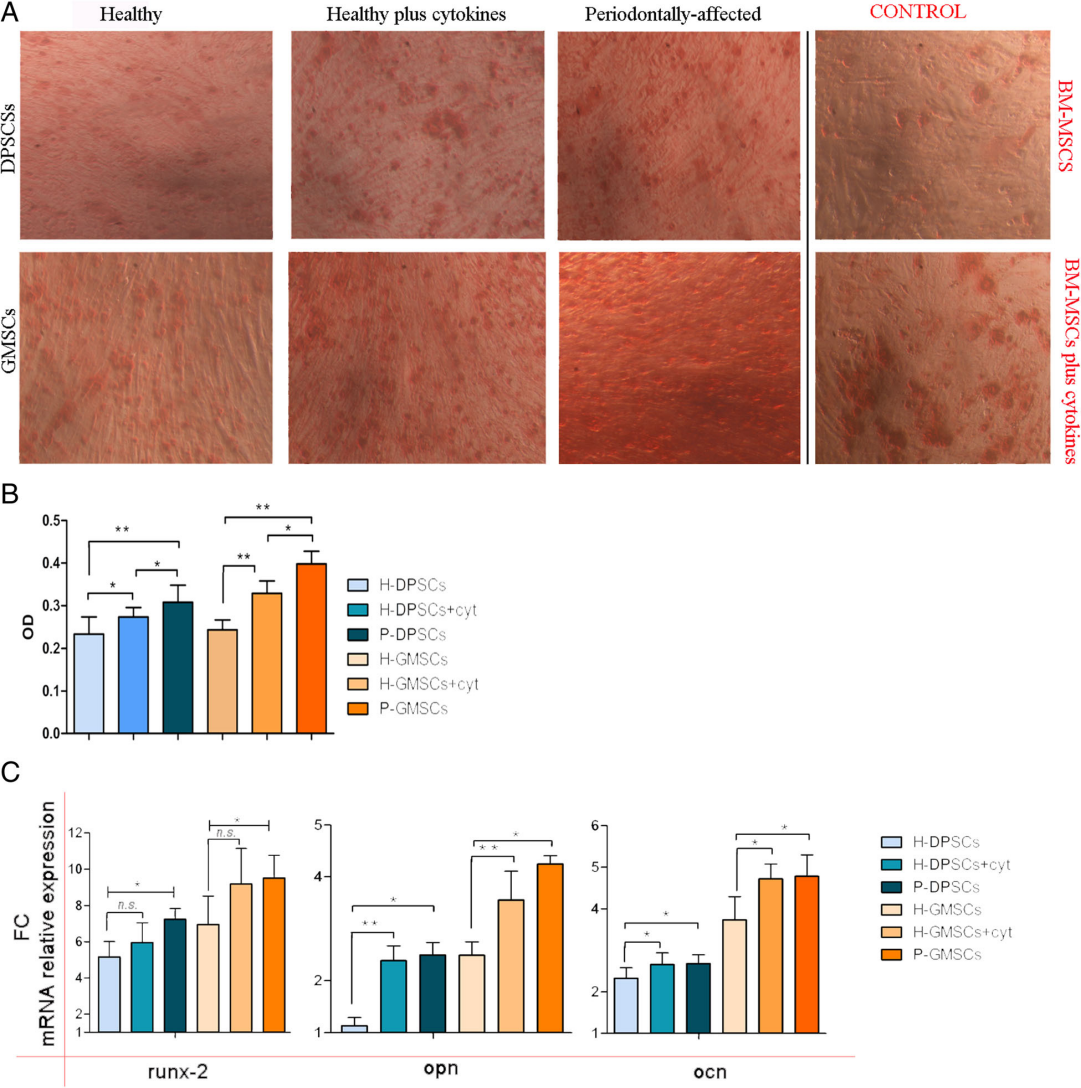
The osteogenic potential of pulpal (*DMSCs*) and gingival (*GMSCs*) mesenchymal stem cells. **a** A representative image of Alizarin Red assay stained calcium deposits after 15 days of osteogenic differentiation cultured in healthy (*H*)-DPSCs and H-GMSCs with or without cytokine (*cyt*) preconditioning, and periodontally affected (*P*)-DPSCs, P-GMSCs, and bone marrow MSCs (*BM-MSCs*) with or without cytokine preconditioning (control). **b** The bar graph represents the quantitative analysis of Alizarin by means of spectrometry (reading at 550 OD). **c** the bar graphs represent the relative mRNA expression of early and later osteogenic differentiation markers. Culture passage P5. *p < 0.05; **p < 0.01. *FC* fold change, *n.s.* not significant

To evaluate the effect of the inflammatory conditions on the osteogenic differentiation potential in vitro, H-DPSCs and H-GMSCs were treated with IL-1β and TNF-α up to 72 h and then they were cultivated in ODM. After 15 days of the differentiation culture procedure, phenotypic and gene analyses were performed to compare treated healthy cells and periodontally affected cells with untreated healthy dental MSCs. Using phase-contrast microscopy the Alizarin Red S staining was evaluated, and we observed that treated MSCs and periodontally affected MSCs appeared more stained than untreated healthy controls (Fig. 6a). Confirming these observations, the Alizarin Red S absorbance was measured at 550 nm (Fig. 6b). We found a relative OD increase of about 1.05 and 1.31 in treated H-DPSCs and P-DPSCs, and of about 1.41 and 1.67 in treated H-GMSCs and P-GMSCs compared to their healthy controls. Moreover, the early osteogenic marker runx-2 was found to be upregulated by 1.41-fold and 1.38-fold, respectively, in P-DPSCs and P-GMSCs (*p* < 0.05) compared to their healthy controls; the difference between treated and untreated MSCs was not significant (*p* > 0.05). However, treated MSCs and periodontally affected MSCs showed significantly higher mRNA levels of the later osteogenic differentiation markers compared to their untreated healthy controls. Specifically, we detected an upregulation in osteopontin levels of 2.09-fold and 2.18-fold in treated H-DPSCs and P-DPSCs and 1.42-fold and 1.71-fold in treated H-GMSCs and P-GMSCs (*p* < 0.05), and upregulation of osteocalcin levels of 1.14-fold and 1.15-fold in treated H-DPSCs and P-DPSCs and 1.25-fold and 1.27-fold in treated H-GMSCs and P-GMSCs (*p* < 0.05) (Fig. 6b).

### IL-1β and TNF-α increase the expression of several ADFs and HSPs

**Fig. 7:**
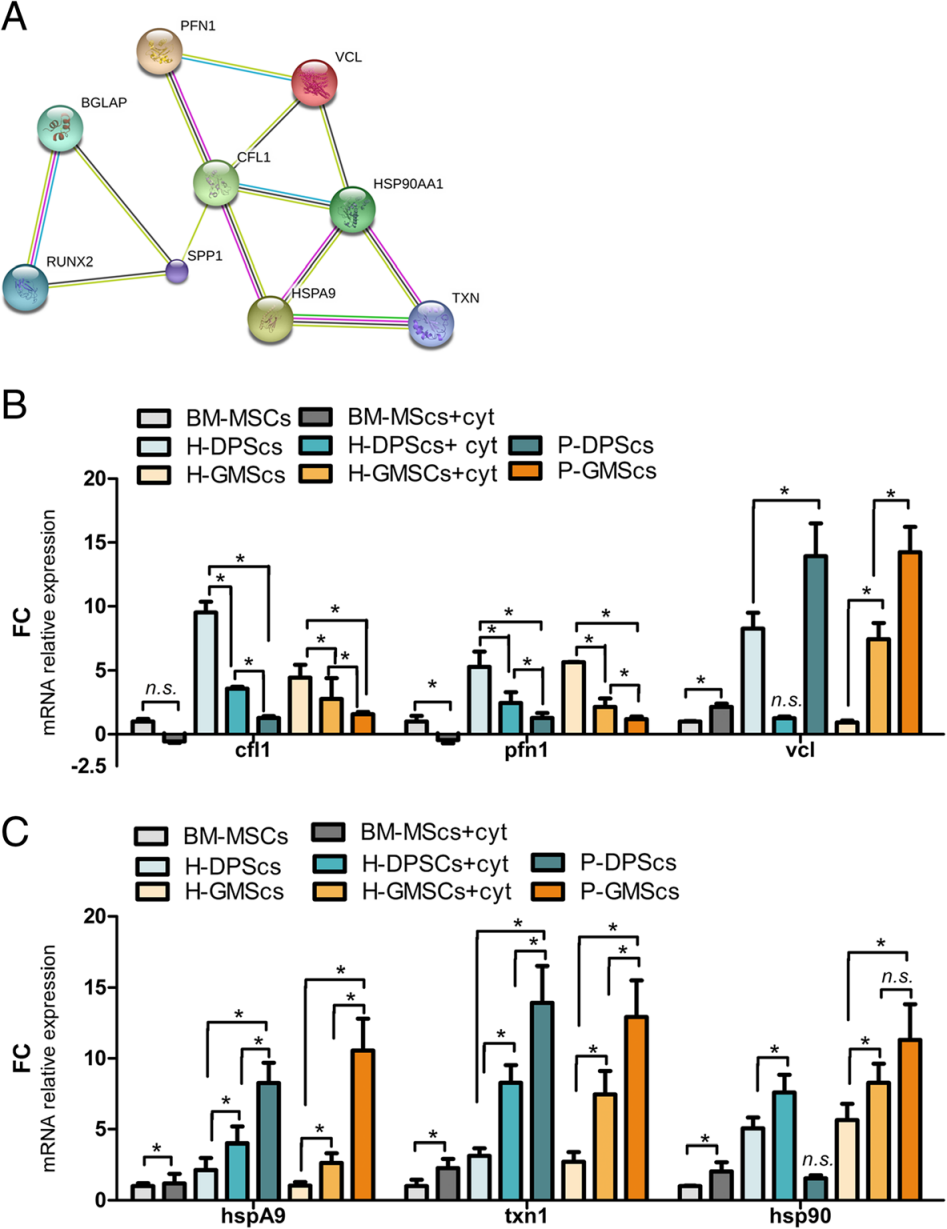
IL-1β and TNF-α increase the expression of several ADFs and HSPs. **a** The interaction between cytoskeleton-regulating proteins, anti-inflammatory chaperone proteins, and the osteogenic specific markers performed at http://string-db.org/. **b**, **c** The bar graphs represent the qPCR analysis for **b** ADF gene and **c** heat shock protein gene in bone marrow mesenchymal stem cells (*BM-MSCs*), BM-MSCs + cytokines (*cyt*; 20 ng/ml IL1β + 40 ng/ml TNFα), healthy (*H*) dental pulp MSCs (*DPSCs*), H-DPSCs + cyt, periodontally affected (*P*)-DPSCs, healthy gingival MSCs (*H-GMSCs*), H-GMSCs + cyt, and P-GMSCs. Mean values ± SD of all samples studied are reported. SD bars are based on three independent experiments. Culture passage P6. *p < 0.05. *FC* fold change, *n.s.* not significant

A computational STRING analysis was performed to investigate the functional interaction between osteogenic differentiation, actin cytoskeletal organization, and cytokine response. The chief members of each protein cluster analyzed were the following: runx-2, opn, ocn (osteogenic differentiation markers), cofilin-1 and profiling-1 (ADFs), vinculin (the key depolymerization factor), and hsp90, hspA9, and txn-1 (the main heat shock proteins). Based on the criteria set, we obtained a network of protein-protein interactions (PPI) that linked together all three protein clusters (Fig. 7a). After 15 days of osteoblastic differentiation, qPCR analysis was assessed to test the mRNA levels of ADFs and HSPs. Generally, the cytokine-treated and periodontally affected MSCs expressed lower mRNA levels of ADFs and higher mRNA levels of HSPs. Specifically, ADF downregulation and vinculin upregulation were found in cytokine-treated and periodontally affected MSCs (*p* < 0.05) with respect to untreated healthy controls (Fig. 7b). Moreover, upper levels of about 2.5- and 5-fold were found in hsp90, hspA9, and txn-1 expression in cytokine-treated and periodontally affected MSCs compared to untreated healthy controls (Fig. 7c).

## Discussion

For decades, extraordinary interest has emerged in the field of MSCs because of their differentiation potential that introduces them for possible use in TE, RM, and cell and gene therapy for clinical applications. Scientists are ongoing in their search for the best source of MSC tissue. The current elective tissues to this end are bone marrow and adipose tissue, although the isolation of these MSCs is an invasive procedure for both patients and donors. In view of this, the possibility to isolate MSCs from discarded tissue is a fascinating idea and MSCs from periodontally affected patients could be a good alternative. Even though it has been shown in the literature that proinflammatory cytokines affect the MSC properties, the effects of inflammation due to periodontitis and its effects on the features of dental MSCs remain unclear [26, 30, 31].

In the present study, we confirmed the presence of MSCs in human dental pulp and gingival tissue harvested from periodontally affected patients and for the first time, to best of our knowledge, we compared their stem features to DPSCs and GMSCs harvested from healthy donors at the same time. The cells isolated from all groups showed a typical fibroblast-like shape and displayed positivity for the principle stem markers Stro-1, CD46, CD29, and SSEA4, and they did not display surface expression for any hematopoietic marker (CD34 and CD45). Our results suggested that the inflamed condition relating to the periodontal status of the patients had no effect on dental MSC viability, whereas it could improve the growth ability and stem cell gene profile of DPSCs and GMSCs. This view was supported by proinflammatory cytokine preconditioning experiments that demonstrated that H-DPSCs and H-GMSCs are able to mimic P-DPSCs and P-GMSCs, displaying similar proliferation curves and gene expressions when treated with IL-1β and TNF-α [31, 32]. Specifically, P-DPSCs and P-GMSCs showed a clear, higher expression of the cardinal stem nuclear markers NANOG, OCT4, and SOX-2, and stem superficial markers ABCG2, CD105, and THY-1, compared to healthy controls and the internal positive control (BM-MSCs). Moreover, our data on H-DPSCs and H-GMSCs under proinflammatory conditions in vitro showed that osteoblastic differentiation capacity is not only well preserved but also is significantly higher than under nonstimulated conditions, and almost equal to periodontally affected MSCs. This evidence was confirmed by a higher calcified extracellular matrix formation and a higher expression of early and later osteogenic differentiation markers (runx2, osteopontin, and osteocalcin) in the cytokine-treated H-DPSCs and H-GMSCs as well as in the periodontally affected MSCs [38–40].

It has already been shown that, in inflammatory environments, several mediators activate a set of biological process such as cell survival, proliferation, and cell differentiation in MSCs [41–46]. During lineage-specific differentiation, human stromal stem cells exhibit significant changes in morphology. The differentiation process is closely linked to the remodeling of the actin cytoskeletal organization by means of the collaboration between the actin depolymerizing factors and chaperone proteins [47–51]. In the first instance, the ADFs bind to actin monomers and filaments, causing depolymerization of actin filaments preventing their reassembly and hindering differentiation [52–55]. In the second instance, chaperone/HSP proteins, including hsp90, hspA9, and thioredoxin, assist the main ADFs (cofilin, profiling, and vinculin) to model actin polymerization status [33–36, 56, 57].

Our in vitro data suggested that the chronically inflamed environment, perpetuated by persistent proinflammatory cytokines, could be an advantage for the human P-DPSCs and P-GMSCs. This inflammatory background maintains high expression levels of hsp90, txn-1, and hspA9 that finally allows the stabilization of actin filaments by means vinculin, profilin-1, and cofilin-1.

## Conclusions

Our results confirm that the chronic inflammatory microenvironment that exists in periodontitis does not negatively affect the number or the stem cell marker profile of P-DPSCs and P-GMSCs. The proinflammatory cytokines permit a higher osteogenic differentiation potential, controlling MSC fate through several regulatory mechanisms involving remodeling of the cytoskeleton and stress response process. This convinces us that periodontally affected MSCs are a valid autologous MSC source and that they could be employed for in vivo applications in diseases with a persistent inflammatory environment that generally harms the features and hinders the success of cell transplantation. From a clinical point of view, these findings are promising for future tissue engineering applications in vivo.

## Additional files

Additional file 1: Figure S1. A) Bar graph represents the MTT absorbance mean values ± SD of P-DPSCs and P-GMSCs vs. their healthy control. The differences were not significant. B) The histograms represent the comparison of DT. Mean values ± SD are based on three independent experiments. n.s = not significant (*p* value > 0.05). C) Each field shows a cell cycle analysis representative of each sample investigated. (TIF 11728 kb)

Additional file 2: Table S1. The expression values of putative mesenchymal surface stem cells markers. (DOC 31 kb)

Additional file 3: Figure S2. A, B) Basal gene expression of IL-1β and TNF-α receptors in P-DPSCs, H-DPSCs, P-GMSCs, and H-GMSCs. C, D) Gene expression of IL-1β and TNF-α receptors under cytokine stimulation at 24, 48, and 72 h (cytokine treatment: 20 ng/ml IL-1β + 40 ng/ml TNF-α). *p value < 0.05; n.s = not significant. FC = fold change. (JPG 239 kb)

## Abbreviations

ABCG2: ATP-binding cassette sub-family G member 2
ADF: Actin depolymerizing factor
BM-MSC: Bone marrow mesenchymal stem cell
CD: Cluster differentiation
Cfl-1: Cofilin-1
DPSC: Dental pulp mesenchymal stem cell
DT: Doubling time
FBS: Fetal bovine serum
f-LSC: Limbal fibroblast-like stem cell
GBR: Guided bone regeneration
GMSC: Gingival mesenchymal stem cell
HSP: Heat shock protein
IL: Interleukin
MSC: Mesenchymal stem cell
NANOG: Homeobox protein NANOG
OCT4: Octamer-binding transcription factor 4
OD: Optical density
ODM: Osteogenic differentiation medium
opn: Osteopontin
P: Passage
PI: Proliferation index
Pfn-1: Profilin-1
qPCR: Quantitative polymerase chain reaction
RM: Regenerative medicine
runx-2: Runt-related transcription factor 2
SOX-2: SRY (sex determining region Y)-box 2
SSEA-4: Stage-specific embryonic antigen-4
STRING: Search Tool for the Retrieval of Interacting Genes/Proteins
TE: Tissue engineering
THY-1: Thy-1 cell surface antigen
TNF: Tumor necrosis factor
Vcl: Vinculin
