# Supplementary figures and images for: Mesenchymal stem cells derived from inflamed dental pulpal and gingival tissue: a potential application for bone formation
Source: Stem Cell Res Ther. 2017 Aug 1;8:179. doi: 10.1186/s13287-017-0633-z (PMC5540218; doi:10.1186/s13287-017-0633-z)

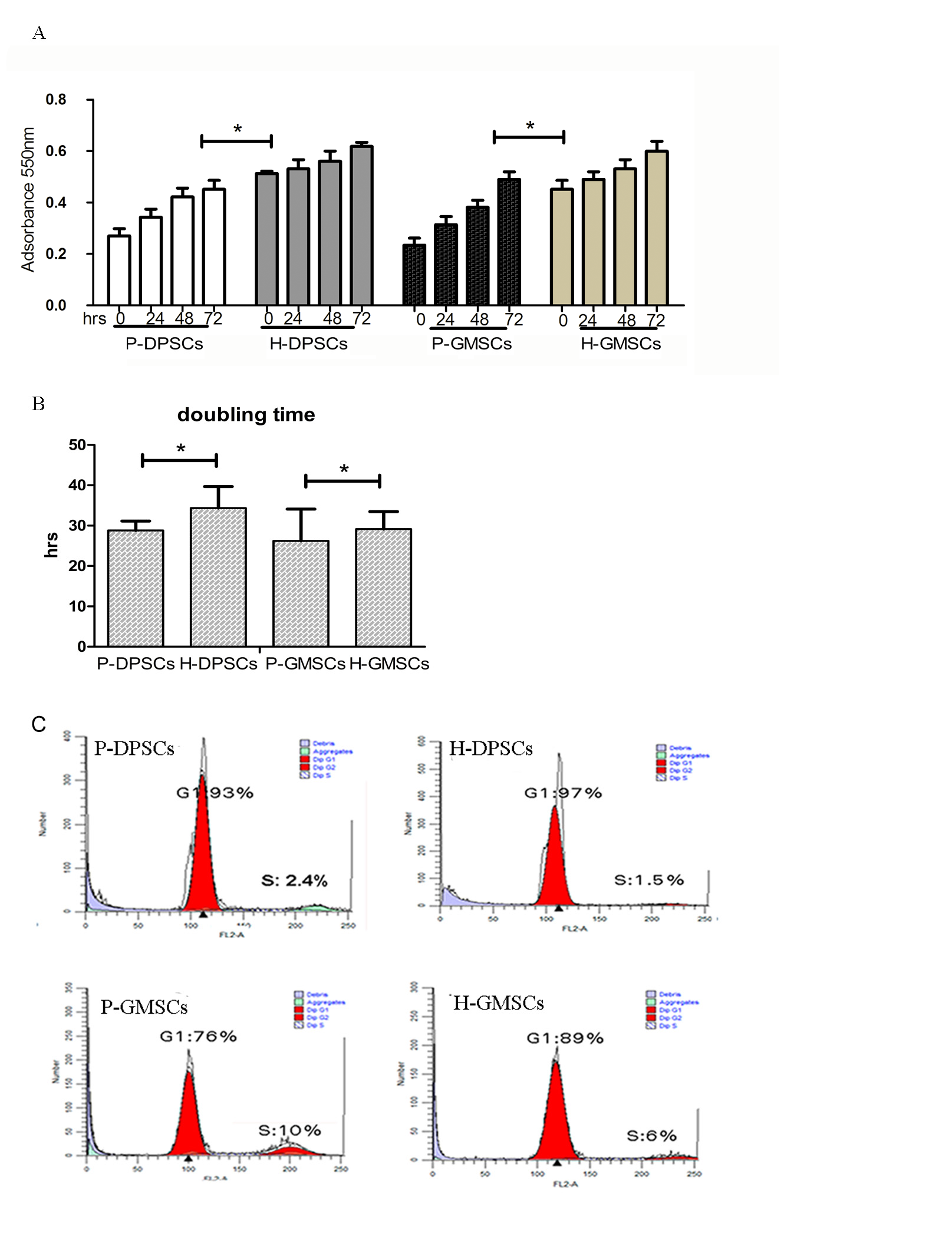

Supplement: Supplementary file 1 — A) Bar graph represents the MTT absorbance mean values ± SD of P-DPSCs and P-GMSCs vs. their healthy control. The differences were not significant. B) The histograms represent the comparison of DT. Mean values ± SD are based on three independent experiments. n.s = not significant (p value > 0.05). C) Each field shows a cell cycle analysis representative of each sample investigated. (TIF 11728 kb) [file 13287_2017_633_MOESM1_ESM.tif]

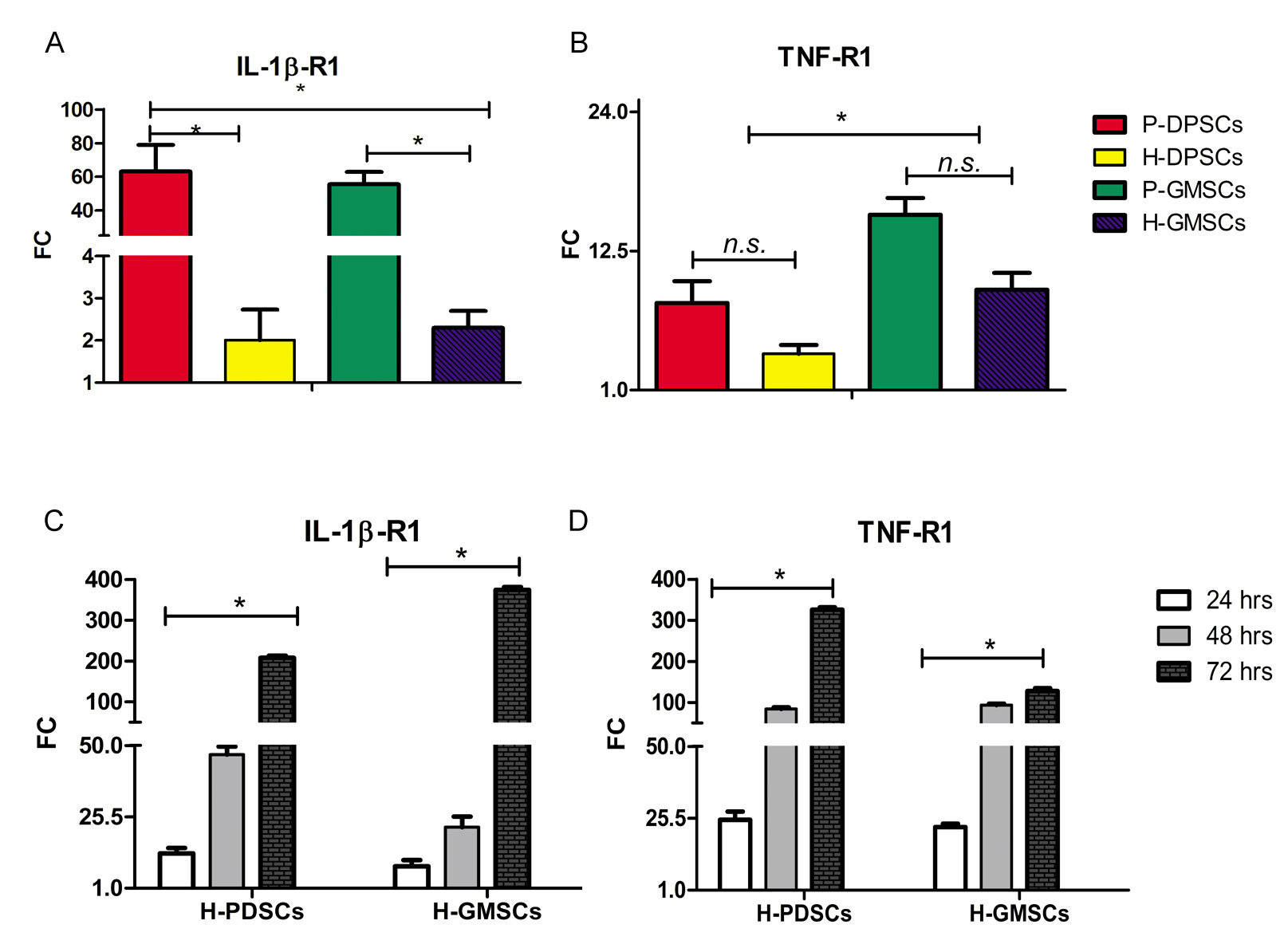

Supplement: Supplementary file 3 — A, B) Basal gene expression of IL-1β and TNF-α receptors in P-DPSCs, H-DPSCs, P-GMSCs, and H-GMSCs. C, D) Gene expression of IL-1β and TNF-α receptors under cytokine stimulation at 24, 48, and 72 h (cytokine treatment: 20 ng/ml IL-1β + 40 ng/ml TNF-α). *p value < 0.05; n.s = not significant. FC = fold change. (JPG 239 kb) [file 13287_2017_633_MOESM3_ESM.jpg]
