# Supplementary material for: Mesenchymal stem cells derived from inflamed dental pulpal and gingival tissue: a potential application for bone formation
Source: Stem Cell Res Ther. 2017 Aug 1;8:179. doi: 10.1186/s13287-017-0633-z (PMC5540218; doi:10.1186/s13287-017-0633-z)
Supplement: Supplementary file 2 — The expression values of putative mesenchymal surface stem cells markers. (DOC 31 kb) [file 13287_2017_633_MOESM2_ESM.doc]

| MSCs | MESENCHYMAL STEM CELL MARKERS | | | |
| --- | --- | --- | --- | --- |
|  | STRO-1 | CD146 | CD29 | SSEA4 |
| P-DPSCs | 31.32 ± 3.32 % | 37.80 ± 4.79 % | 97.26 ± 2.38 % | 27.33 ± 2.71 % |
| H-DPSCs | 12.30 ± 2.32 % | 18.95 ± 1.79 % | 99.07 ± 0.5 % | 18.31 ± 1.06 % |
| P-GMSCs | 20.46 ± 2.18 % | 86.54 ± 3.76 % | 96.74 ± 3.68 % | 31.56 ± 2.54 % |
| H-GMSCs | 10.61 ± 1.37 % | 73.34 ± 5.43 % | 96.64 ± 5.08 % | 17.72 ± 1.97 % |
| BM-MSCs | 6.25 ± 2.2 % | 44.89 ± 5.54 % | 97.21 ± 0.89% | 5.1 ± 1.53 % |

Table S1 The MSC marker level expressions in healthy and periodontally-affected DPSCs or GMSCs. Standard deviations (±).
